# Supplementary material for: Committee machines—a universal method to deal with non-idealities in memristor-based neural networks
Source: Nat Commun. 2020 Aug 26;11:4273. doi: 10.1038/s41467-020-18098-0 (PMC7450095; doi:10.1038/s41467-020-18098-0)
Supplement: Supplementary file 1 — Supplementary Information [file 41467_2020_18098_MOESM1_ESM.pdf]

Supplementary Information for the Paper
"Committee Machines—A Universal Method to Deal with Non-Idealities in Memristor-Based Neural Networks"

Joksas et al.

SUPPLEMENTARY FIGURES

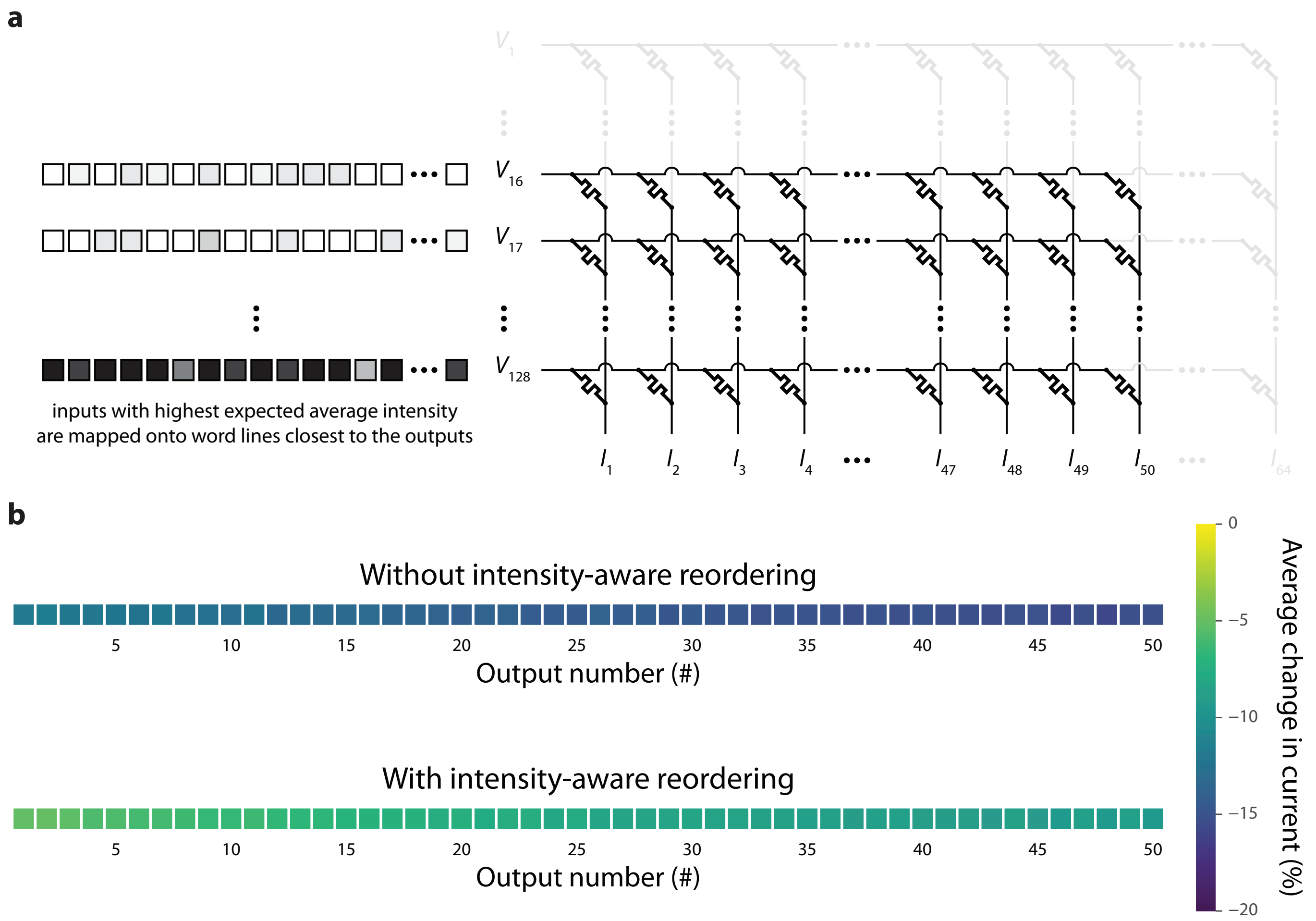

Supplementary Figure 1: **Implementation of intensity-aware reordering in crossbar arrays. a** Mapping the first subset of inputs onto word lines of one of the seven Ta/HfO_2 crossbars (of shape 128×64) used to implement the whole synaptic layer of shape 785×25. **b** Heatmap of average changes in output currents due to line resistance (in all seven crossbars) without and with intensity-aware reordering of the inputs. For this particular simulation, it was assumed that Ta/HfO_2 devices can be programmed perfectly.

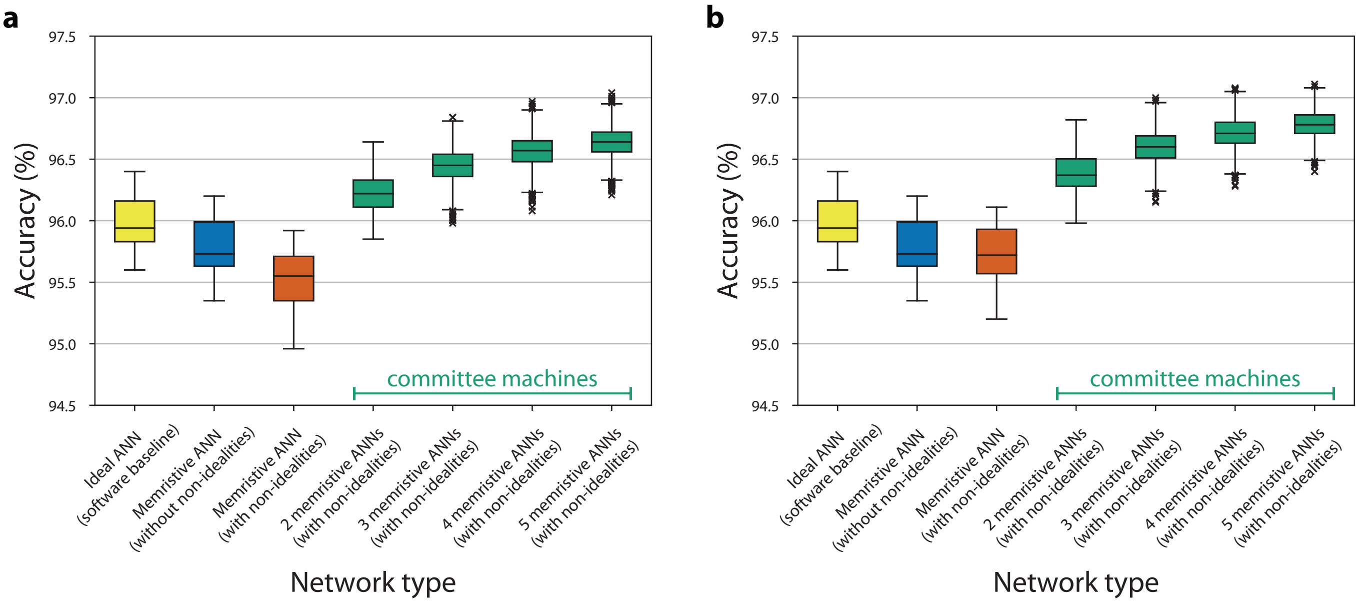

Supplementary Figure 2: **Performance of intensity-aware reordering when dealing with low line resistance.** Box plots show the accuracy of networks that were disturbed using interconnect resistance from Ta/HfO_2 crossbar. **a** Without intensity-aware reordering. **b** With intensity-aware reordering. In both box plots, the maximum whisker length is set to $1.5 \times$ IQR.

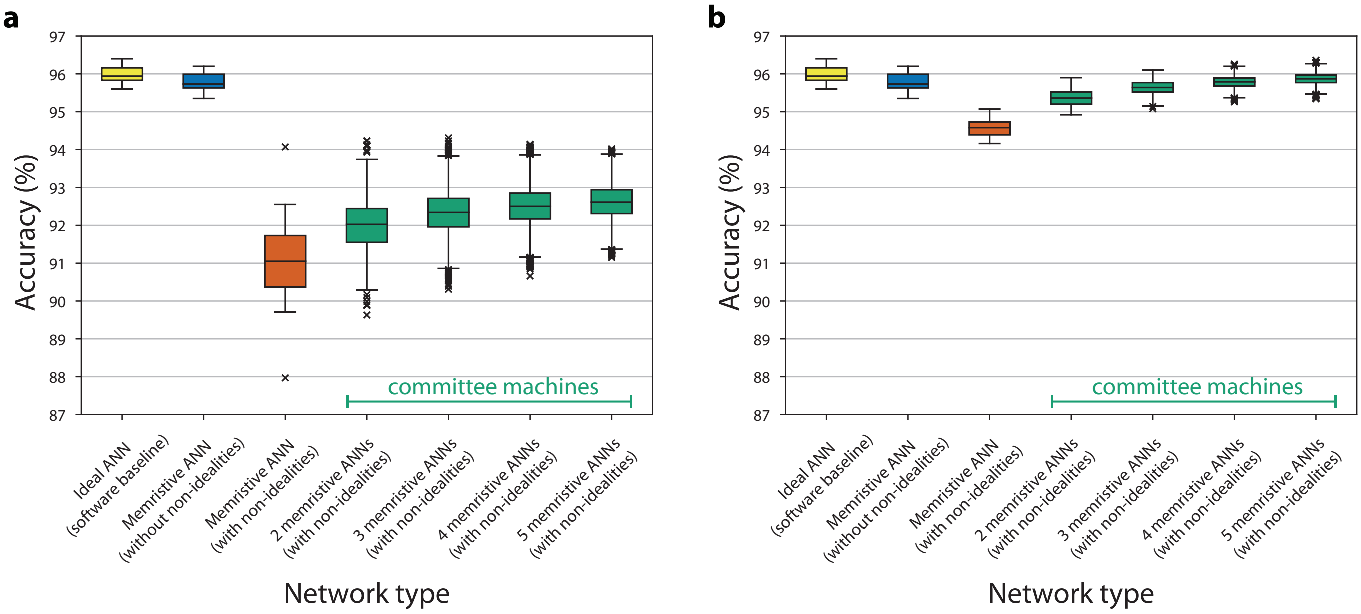

Supplementary Figure 3: **Performance of intensity-aware reordering when dealing with high line resistance.** Box plots show the accuracy of networks that were disturbed using interconnect resistance that is 5 times higher than the one from Ta/HfO_2 crossbar. **a** Without intensity-aware reordering. **b** With intensity-aware reordering. In both box plots, the maximum whisker length is set to $1.5 \times$ IQR.

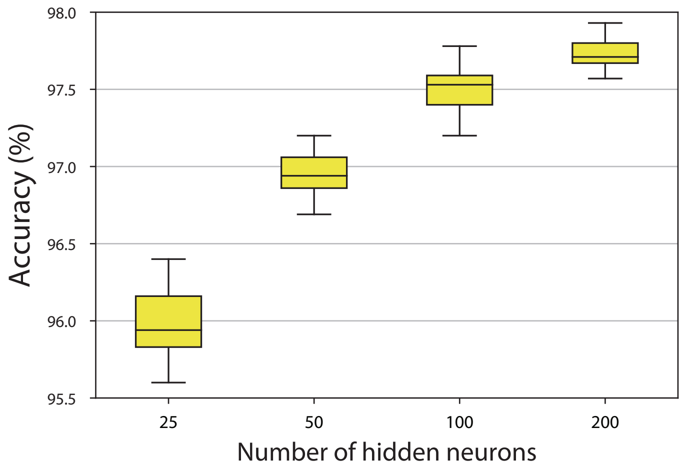

Supplementary Figure 4: **Accuracy of digitally implemented networks containing one hidden layer.** Accuracy is shown for different number of hidden neurons. In the box plot, the maximum whisker length is set to 1.5 × IQR.

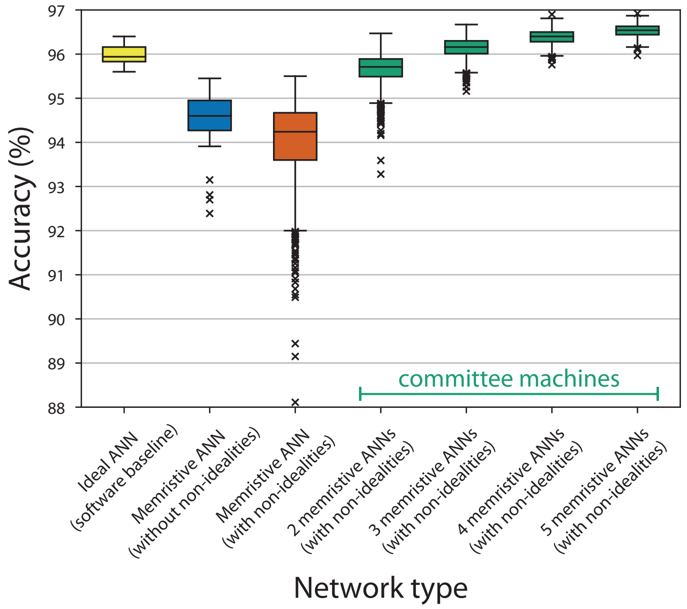

Supplementary Figure 5: **Effectiveness of committees with numerically optimised weightings.** Networks were disturbed using RTN data from Ta_2O_5 device. In the box plot, the maximum whisker length is set to 1.5 × IQR.

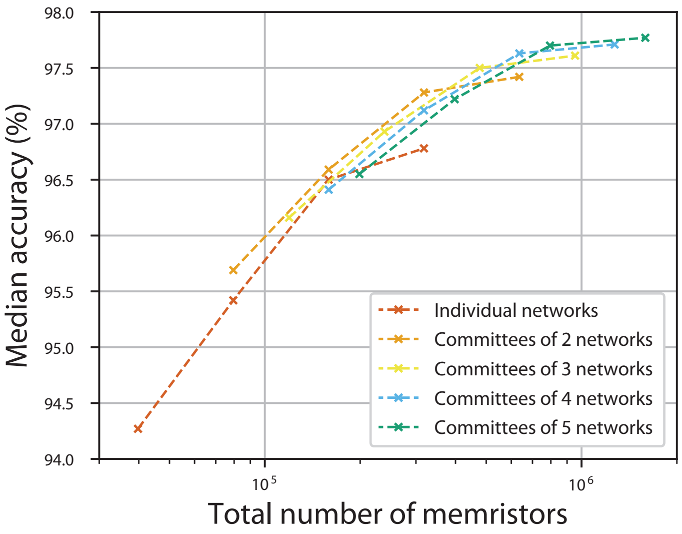

Supplementary Figure 6: **Effectiveness of committee machines when controlling for the total number of Ta_2O_5 devices.** Median accuracy achieved by individual one-hidden-layer memristor-based networks and their committees. The networks contained 25, 50, 100 or 200 hidden neurons and were disturbed using RTN data from a Ta_2O_5 device.

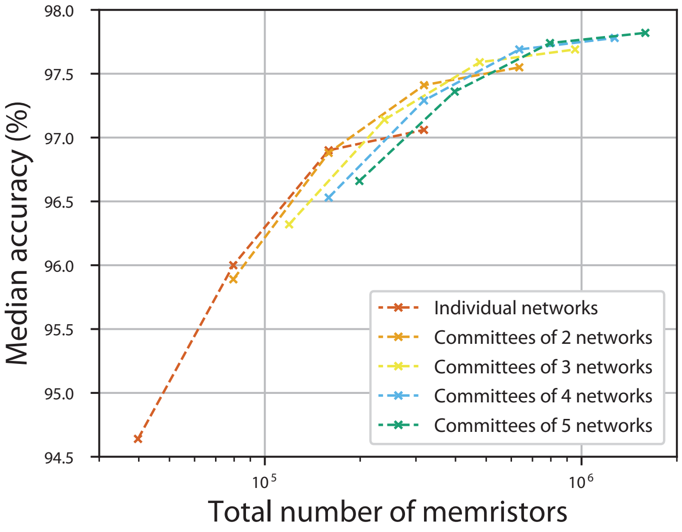

Supplementary Figure 7: **Effectiveness of committee machines when controlling for the total number of aVMCO devices.** Median accuracy achieved by individual one-hidden-layer memristor-based networks and their committees. The networks contained 25, 50, 100 or 200 hidden neurons and were disturbed using RTN data from an aVMCO device.

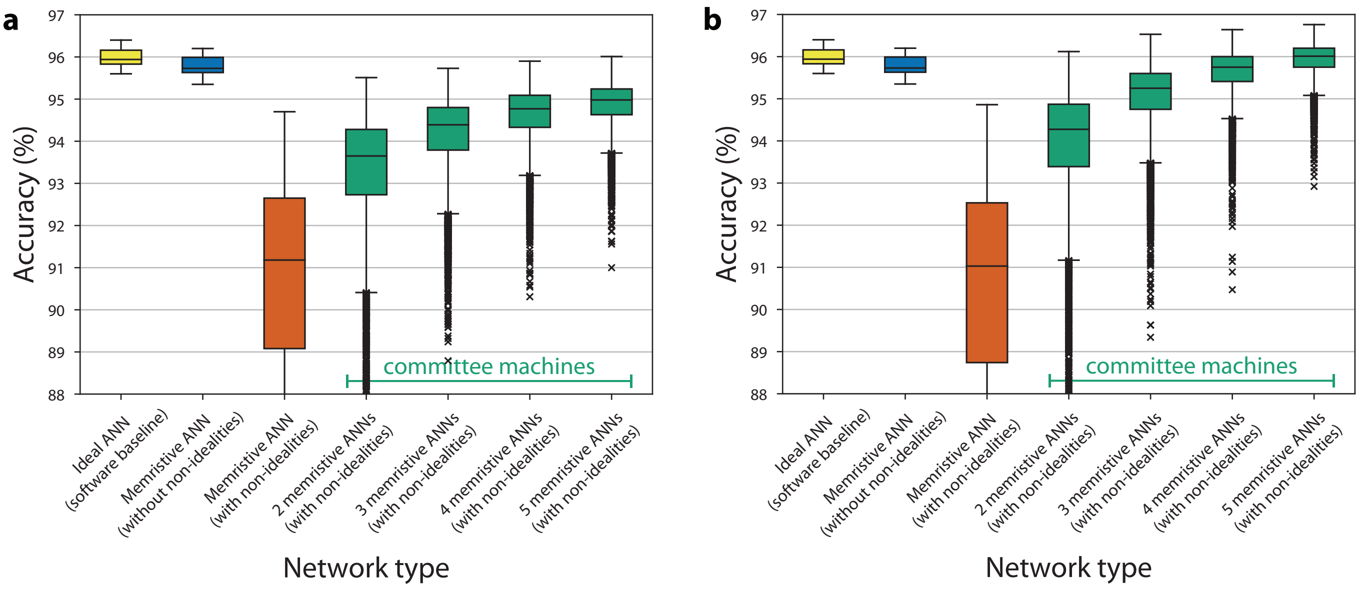

Supplementary Figure 8: **Comparison of methods of constructing committee machines.** Accuracy achieved by individual networks and their committees when faulty devices and D2D variability data of Ta/HfO_2 crossbar are taken into account. **a** Using identical digital networks when implementing committees of memristive neural networks. **b** Using different digital networks when implementing committees of memristive neural networks. The maximum whisker length in both subfigures is set to $1.5 \times$ IQR. The accuracy of individual disturbed non-ideal memristive networks in the two subfigures is not *identical* only because the data were produced using two different simulations. However, it is clear that using different digital networks results in higher accuracy of the committees.

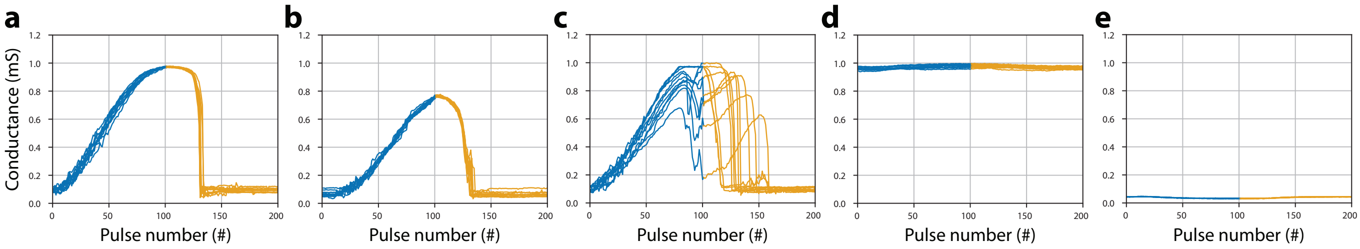

Supplementary Figure 9: **Pulsing data from Figures 2b-f represented in Cartesian form.** 11 SET cycles are depicted in blue, while 11 RESET cycles are depicted in orange. **a** Equivalent of Figure 2b. **b** Equivalent of Figure 2c. **c** Equivalent of Figure 2d. **d** Equivalent of Figure 2e. **e** Equivalent of Figure 2f.

SUPPLEMENTARY TABLES

**Figures**	**Device type**	**HRS/LRS**	**Number of conductance states**	**Spacing of states**	**Network architecture**	p_L **(%)**
3b, 4, 9, 2(S), 3(S), 8(S)	Ta/HfO_2	10.48	∞	-	784(+1):25(+1):10	0.1
6, 5(S), 6(S)	Ta_2O_5	8	8	Equally spaced resistance states	784(+1):25(+1):10	0.1
8, 7(S)	aVMCO	7.5	8	{1.00 MΩ, 1.92 MΩ, 2.84 MΩ, 3.76 MΩ, 4.68 MΩ, 5.60 MΩ, 6.52 MΩ, 7.50 MΩ} (nearly equally spaced resistance states)	784(+1):25(+1):10	0.1
9	Ta/HfO_2	10.48	∞	-	784(+1):50(+1):10	0.0
9	Ta/HfO_2	10.48	∞	-	784(+1):100(+1):10	0.1
9	Ta/HfO_2	10.48	∞	-	784(+1):200(+1):10	0.0
6(S)	Ta_2O_5	8	8	Equally spaced resistance states	784(+1):50(+1):10	0.1
6(S)	Ta_2O_5	8	8	Equally spaced resistance states	784(+1):100(+1):10	0.1
6(S)	Ta_2O_5	8	8	Equally spaced resistance states	784(+1):200(+1):10	0.0
7(S)	aVMCO	7.5	8	{1.00 MΩ, 1.92 MΩ, 2.84 MΩ, 3.76 MΩ, 4.68 MΩ, 5.60 MΩ, 6.52 MΩ, 7.50 MΩ} (nearly equally spaced resistance states)	784(+1):50(+1):10	0.1
7(S)	aVMCO	7.5	8	{1.00 MΩ, 1.92 MΩ, 2.84 MΩ, 3.76 MΩ, 4.68 MΩ, 5.60 MΩ, 6.52 MΩ, 7.50 MΩ} (nearly equally spaced resistance states)	784(+1):100(+1):10	0.1
7(S)	aVMCO	7.5	8	{1.00 MΩ, 1.92 MΩ, 2.84 MΩ, 3.76 MΩ, 4.68 MΩ, 5.60 MΩ, 6.52 MΩ, 7.50 MΩ} (nearly equally spaced resistance states)	784(+1):200(+1):10	0.4

Supplementary Table 1: Summary of parameters for each simulation in the main text and supplementary information. Infinite number of states simply means that, during the mapping of weights onto pairs of conductances, the inability to program the devices precisely is not taken into account, only their HRS/LRS ratio is. However, the imprecision in programming can be taken into account during disturbance stage, as was done with Ta/HfO_2 memristors. Supplementary figures are followed by "(S)".

**Resistance level**	25 kΩ	50 kΩ	75 kΩ	100 kΩ	125 kΩ	150 kΩ	175 kΩ	200 kΩ
**RTN occurrence rate**	40.625%	43.75%	46.875%	59.375%	62.5%	65.625%	68.75%	71.875%

Supplementary Table 2: Occurrence rate of RTN in Ta_2O_5 device.

**Resistance level**	1.00 MΩ	1.92 MΩ	2.84 MΩ	3.76 MΩ	4.68 MΩ	5.60 MΩ	6.52 MΩ	7.50 MΩ
**RTN occurrence rate**	6.67%	8.89%	8.89%	15.6%	20%	20%	24.4%	28.9%

Supplementary Table 3: Occurrence rate of RTN in aVMCO device.

SUPPLEMENTARY NOTES

Supplementary Note 1

As discussed in the main text, high interconnect resistance can significantly reduce the accuracy of physically implemented ANNs. Large current decreases at the outputs often result from large input voltages that are applied at the top part of the crossbar, far away from the outputs. Such inputs generate large amounts of current that flow through large portions of the bit lines and, with voltage drops across interconnects, disturb the overall current distribution in a major way.

In some applications, such as supervised learning, it might be possible to strategically map certain inputs to certain word lines, so that the effect of line resistance is minimised. We propose intensity-aware reordering in which ANN's inputs with highest expected average intensities are mapped onto word lines closest to the outputs of a crossbar. This makes it so that most of the current is generated near the outputs, while the currents in the top parts of the bit lines are disturbed minimally. The key challenge here is to predict which inputs would have the highest average intensities. In supervised learning, this can be done by recording the average intensities over training and verification sets (if these sets are truly representative of the test set).

Supplementary Figure 1a shows how the inputs would be reordered so that the ones with highest expected average intensity would be placed nearest to the outputs. Following the example from Figure 3b in the main text, Supplementary Figure 1b demonstrates the effectiveness of intensity-aware reordering in reducing current decreases. Without the reordering scheme, current decreases due to line resistance ranged from ∼12% to ∼16%, while with the intensity-aware reordering, current decreases range from ∼5% to ∼9%.

Supplementary Figure 2 shows a comparison of accuracies achieved by individual networks and their committees without and with intensity-aware mapping. Without intensity-aware reordering, the accuracy of individual non-ideal memristive networks drops to ∼95.6% and in committees of 5, it increases to ∼96.6%. With intensity-aware mapping, the accuracy in individual non-ideal networks drops only to ∼95.7%—effectively the same accuracy as of individual memristive networks without the non-idealities. In committees of 5, the accuracy goes up to ∼96.8%.

Because the interconnect resistance of Ta/HfO_2 crossbar is relatively low, the line resistance effects in Supplementary Figure 2 are not very severe. To test the limits of intensity-aware mapping and CM method, we performed an additional simulation that involved five times higher interconnect resistance (1.75 Ω along the word lines and 1.6 Ω along the bit lines). The equivalent comparison with this higher interconnect resistance is shown in Supplementary Figure 3. Here we can clearly see the effectiveness of intensity-aware reordering because using it yields ∼94.6% accuracy in individual non-ideal memristive networks, compared to ∼91.0% in networks not using this scheme. However, Supplementary Figure 3 also demonstrates the limits of the CM method. In Supplementary Figure 3a, the accuracy in committees of 5 increases only up to ∼92.6%, indicating that committees struggle with large changes in current due to line resistance. This might be due to the uni-directional nature of line resistance—in all crossbars, the currents tend to decrease, meaning that different networks in a committee could struggle to compensate for each other's non-idealities.
